# Experimental Study on Grouting Seepage Characteristics in Rough Single Microfissure Under Triaxial Stress States

**DOI:** 10.3390/ma18163746

**Published:** 2025-08-11

**Authors:** Minghao Yang, Shuai Zhang, Mingbin Wang, Junling Qin, Wenhan Fan, Yue Wu

**Affiliations:** 1 School of Hydraulic and Civil Engineering, Ludong University, Yantai 264025, China; ymh0214678@163.com (M.Y.); wangmingbin@ldu.edu.cn (M.W.); 2 Shandong Key Laboratory of Civil Engineering Disaster Prevention and Mitigation, Shandong University of Science and Technology, Qingdao 266590, China; junlingqin92@163.com (J.Q.); wysdkd2019@sdust.edu.cn (Y.W.); 3 Yantai Municipal Conservation Center, Yantai 264025, China; wenhanfan233@163.com

**Keywords:** sandstone-like microfissures, ultrafine cement slurry, grout diffusion, joint roughness coefficient

## Abstract

The increasing depth of coal mine construction has led to complex geological conditions involving high ground stress and elevated groundwater levels, presenting new challenges for water-sealing technologies in rock microfissure grouting. This study investigates ultrafine cement grouting in microfissures through systematic analysis of slurry properties and grouting simulations. Through systematic analysis of ultrafine cement grout performance across water–cement (W/C) ratios, this study establishes optimal injectable mix proportions. Through dedicated molds, sandstone-like microfissures with 0.2 mm apertures and controlled roughness (JRC = 0–2, 4–6, 10–12) were fabricated, and instrumented with fiber Bragg grating (FBG) sensors for real-time strain monitoring. Triaxial stress-permeation experiments under 6 and 7 MPa confining pressures quantify the coupled effects of fissure roughness, grouting pressure, and confining stress on volumetric flow rate and fissure deformation. Key findings include: (1) Slurry viscosity decreased monotonically with higher W/C ratios, while bleeding rate exhibited a proportional increase. At a W/C ratio = 1.6, the 2 h bleeding rate reached 7.8%, categorizing the slurry as unstable. (2) Experimental results demonstrate that increased surface roughness significantly enhances particle deposition–aggregation phenomena at grouting inlets, thereby reducing the success rate of grouting simulations. (3) The volumetric flow rate of ultrafine cement grout decreases with elevated roughness but increases proportionally with applied grouting pressure. (4) Under identical grouting pressure conditions, the relative variation in strain values among measurement points becomes more pronounced with increasing roughness of the specimen’s microfissures. This research resolves critical challenges in material selection, injectability, and seepage–deformation mechanisms for microfissure grouting, establishing that the W/C ratio governs grout performance while surface roughness dictates grouting efficacy. These findings provide theoretical guidance for water-blocking grouting engineering in microfissures.

## 1. Introduction

The exploitation of deep mineral resources constitutes a critical foundation for global sustainable resource development. With the depletion of shallow deposits, mining operations progressively extend to deeper strata characterized by elevated geostress conditions [1,2,3,4]. At increased depths, roadway-surrounding rocks develop significant stress concentrations. Prolonged high geostress induces plastic deformation and microfissure networks in coal–rock masses, which not only compromise structural integrity and mechanical strength but also establish preferential groundwater seepage pathways. This synergistic degradation mechanism accelerates surrounding rock deterioration, leading to roadway deformation, support failure, and substantial safety risks [5]. Furthermore, mining-induced stresses and subsequent excavation disturbances provoke secondary fracturing in microfissured rock masses, progressively deteriorating the structural and mechanical properties of deep roadway peripheries until catastrophic instability occurs [6,7,8].

Grouting reinforcement has emerged as a prevalent methodology for enhancing microfissured rock integrity, widely implemented in underground engineering to mitigate water seepage through microfissures [9]. The performance of grouting materials fundamentally governs reinforcement efficacy. Ideal grouts require optimal fluidity, penetrability, rapid setting, and high early strength. Conventional cement-based grouts, despite their abundance and cost-effectiveness, exhibit limitations including low stone formation rate, coarse particle size, prolonged curing, and autogenous shrinkage, rendering them inadequate for deep engineering applications [10]. Ultrafine cement grouts demonstrate rheological properties comparable to chemical grouts while maintaining cementitious advantages [11,12,13]. Recent studies reveal: Sun et al. [14] documented enhanced rheology and compressive strength in grouts incorporating basalt powder and fly ash; Guan et al. [15] employed fly ash and a foaming agent to reinforce shield muck, observing that the fly ash effectively filled internal pores within the stone matrix, thereby enhancing its strength; Guo et al. [16] developed coal gangue-fly ash-bentonite composites exhibiting superior impermeability; Kaufmann et al. [17] demonstrated that blending ordinary Portland cement with ultrafine cement improves the rheological properties of grout and enhances the compressive and flexural strength of grouting materials; Guo et al. [18] demonstrated naphthalene superplasticizers improve cementitious matrix densification.

The dynamic transport of cementitious grouts within rock microfissures remains a critical challenge in geotechnical engineering. Pioneering studies have advanced grouting simulation methodologies: Ding et al. [19] developed a visual grouting system integrated with COMSOL 6.0 simulations using a modified Bingham–Papanastasiou model, achieving <9.6% deviation in pressure field predictions; Du et al. [20] demonstrated tortuous microfissures (fractal dimension > 1.1) reduce far-field sealing efficiency by 15% despite 30% near-inlet improvement under low flow rates (<0.1 m/s); Song et al. [21] quantified viscosity sensitivity to mixing speed, revealing ultrafine cement exhibits 2.3× higher rheological variation than OPC; Liu et al. [22] optimized grout formulations via RSM, identifying peak performance at W/C = 0.8 with 40% clay and 1.5% nano-CaCO_3_. Lei et al. [23] demonstrated that increased roughness reduces initial steady seepage and shortens stabilization time in concrete microfissures. Liu et al. [24] conducted a series of dynamic grouting tests with different grouting parameters and found that the microfissure roughness had a significant effect on the grouting effect. Xu et al. [25] used X-ray CT to reveal staged permeability decline due to particle deposition in irregular microfissures. Niu et al. [26] complements this by proving polydisperse particle size dictates clogging patterns (large particles accelerate blockage; small particles reduce hydraulic conductivity at depth). Liu et al. [27] showed grout-filled cracks effectively suppress stress concentration, and Zhang et al. [28] quantified pre-failure permeability reduction of microfissured rock under triaxial compression. These advancements highlight the necessity to couple flow velocity, material properties, and microfissure geometry. However, critical gaps persist: (1) oversimplified parallel-plate models neglect roughness spatial heterogeneity; (2) insufficient integration of grout–microfissure interaction mechanisms.

Nowadays, researchers have focused considerable attention on the scientific research and engineering experiments of ultrafine cement-based slurries, leading to the accumulation of substantial findings. However, there is a relative lack of research focusing on the grouting and seepage law of ultrafine cement-based grouting materials in microfissures of rock bodies. In view of this, this study firstly analyzed the effect of the W/C ratio as the main factor on the viscosity and water precipitation rate of ultrafine cement slurry, theoretically analyzed the injectability of ultrafine cement slurry, and determined the matching ratio of ultrafine cement slurry used in the grouting test. Secondly, a set of special molds was made by preparing a specimen that can monitor the microfissure seepage, and three kinds of sandstone microfissure grouting specimens with an initial opening of 0.2 mm and roughnesses of JRC = 0–2, JRC = 4–6, and JRC = 10–12 were prepared for the grouting test, and a fiber-optic grating sensor was pre-buried in the interior of the specimen as a way of monitoring. Finally, using the microfissure triaxial stress grouting seepage model test system, under the conditions of peripheral pressure of 6 MPa and 7 MPa, respectively, the three kinds of sandstone microfissure specimens with different roughness were subjected to the simulation test of ultrafine cement slurry grouting under different grouting pressures, and the volumetric flow rate of slurry in the specimens with different roughness at various stages was recorded, and the fiber-optic grating sensors were used to monitor the changes of slurry on the microfissure surface under different grouting conditions, which was based on the fiber-optic grating sensor. At the same time, fiber-optic grating sensors were used to monitor the changes of the slurry on the microfissure surfaces under different grouting conditions, and the effects of roughness, grouting pressure, and peripheral pressure on the grouting pattern of sandstone microfissures were analyzed based on the test results.

## 2. Materials and Methods

### 2.1. Materials

#### 2.1.1. Ultrafine Cement

**Figure 1:**
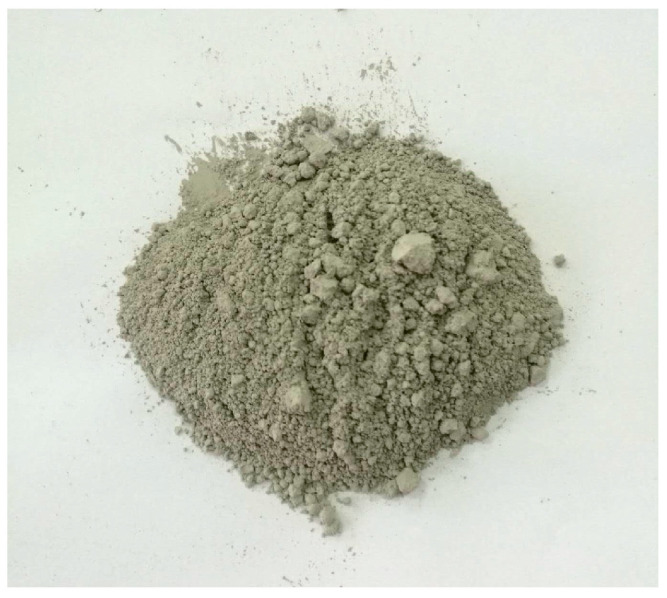
K1340 ultrafine Portland cement.

The experimental study utilized industrial-grade K1340 ultrafine Portland cement sourced from Shandong Province, with microstructural characteristics as characterized in Figure 1.

Its physical and mechanical properties are presented in Table 1.

#### 2.1.2. Sandstone-like Material

**Figure 2:**
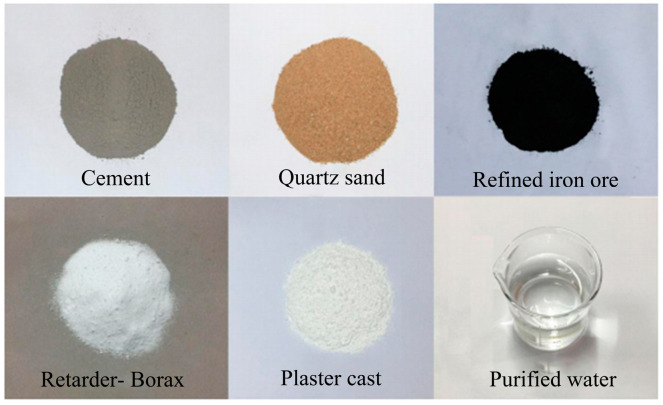
Test materials.

Song Weijie et al. [29] proposed a primary composition scheme for microfissured sandstone simulant materials, utilizing sand and iron concentrate powder as aggregates and cement and gypsum as cementing materials. Through orthogonal experimental methodology, the research team established the optimal mix ratio: an aggregate-to-cementitious material ratio of 7:3 to ensure effective synergism between skeletal structures and binders; a sand-to-iron powder ratio of 2:1 to optimize internal structural composition of aggregates; a cement-to-gypsum ratio of 3:1 to regulate cementitious material performance; and a water content of 25% of the total mass. Experimental materials are illustrated in Figure 2.

The physical and mechanical properties of the ordinary Portland cement used in the experiments are presented in Table 2.

### 2.2. Preparation of Sandstone-like Specimens

#### 2.2.1. Mold Preparation

**Figure 3:**
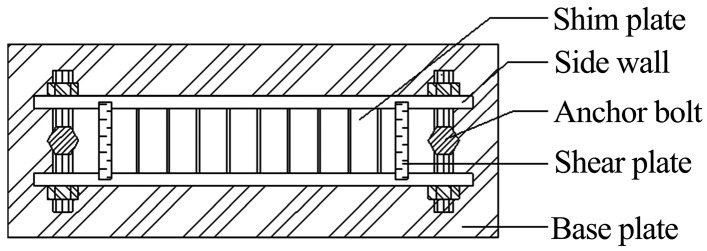
Schematic of Assembly 1 mold structure.

**Figure 4:**
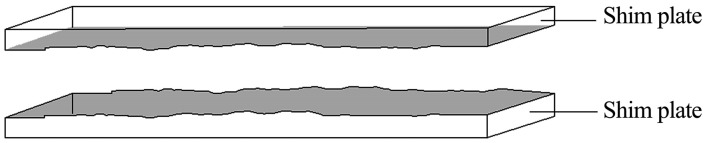
Laser-engineered spacer plates with Barton JRC profiles.

**Figure 5:**
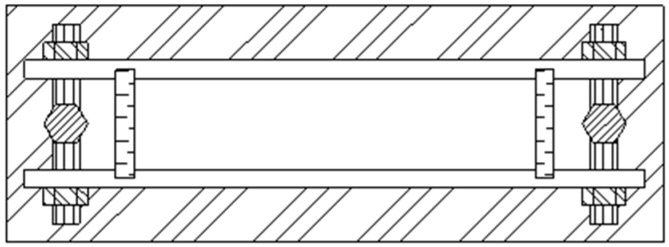
Schematic of Assembly 2 mold structure.

**Figure 6:**
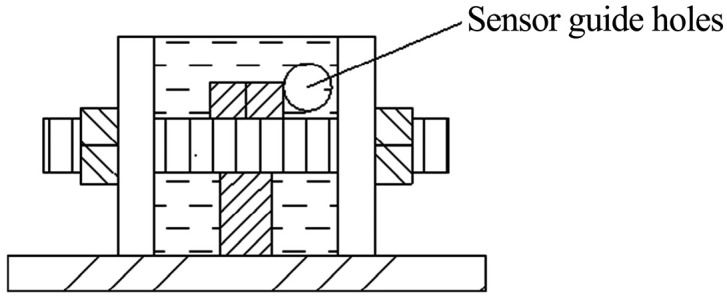
Lateral view of Assembly 2 highlighting sensor conduits.

The methodology for manufacturing groutable sandstone-analog specimens with monitorable microfissure networks employs two modular mold assemblies (three molds per set), with structural configurations detailed in Figure 3, Figure 4, Figure 5 and Figure 6.

The first set of molds primarily consists of base plates, insert plates, fixed bolts, side walls, and spacer plates. The first set of molds is paired as a set, differing in the configuration of internal spacer plates.

A pair of spacer plates is depicted in Figure 4. Utilizing laser wire cutting technology, digitized coordinates of the JRC standard roughness curve were input into a CNC wire-cut machine to precisely fabricate the Barton standard contour profile on steel plate surfaces. This process achieved quantifiable surface roughness, with coupled curves formed on both steel plates.

The second set of molds comprises base plates, insert plates, fixed bolts, and side walls. These conduits enable routing of FBG sensor cables while maintaining structural integrity under triaxial loading.

All components of both mold sets are constructed from high-strength steel to eliminate deformation-induced experimental errors.

#### 2.2.2. Fabrication Process of Sandstone-like Specimens

The fabrication of grouting test blocks with microfissures in sandstone involves three primary steps: (1) preparation of initial specimens; (2) fabrication of core specimens; (3) casting of grouting test blocks containing microfissured sandstone.

Detailed methods for the preparation of monitorable microfissure grouting specimens are described below:

(1) Preparation of cement mortar: the cement mortar required for the experiment was formulated according to specified mix proportions.
(2) Mold preparation: the inner surfaces of Mold 1-1 and Mold 1-2 were uniformly coated with a layer of release agent.
(3) Core specimen fabrication: the mortar was vibrated and compacted in the molds; the upper surfaces were finished, and the specimens were cured for 7 days to complete Specimens 1-1 and 1-2.
(4) Optical fiber integration: a groove was machined on the roughened surface of Specimen 1-1; an optical fiber segment with FBG sensing points was positioned within the groove and secured.
(5) Specimen assembly: specimen 1-2 was placed onto the processed Specimen 1-1, ensuring precise contour matching to form the core specimen assembly.
(6) Gap control and sealing: thin steel shims were inserted into the gaps at both ends of the core specimen; lateral gaps on the specimen surface were sealed.
(7) Secondary mold setup: the core specimen assembly was placed into Mold 2. Exposed fiber-optic leads were guided out through designated ports.
(8) Concrete encapsulation: concrete slurry was poured into Mold 2, followed by vibration, compaction, and surface finishing.
(9) Demolding and shim removal: after 24 h of curing, Mold 2 was removed. The pre-inserted steel shims at both ends were extracted.
(10) Final curing: curing continued for 28 days to complete the permeable test specimen with integrated monitoring capability.

The specific implementation is exemplified by the fabrication process of a sandstone-like grouting test block containing microfissures with an aperture of 200 μm and roughness coefficient JRC = 10–12. The external dimensions of the grouting test block are 300 × 100 × 100 mm.

**Figure 7:**
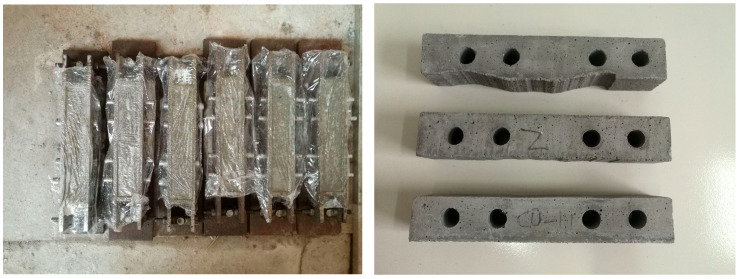
Base matrix fabrication process.

**Figure 8:**
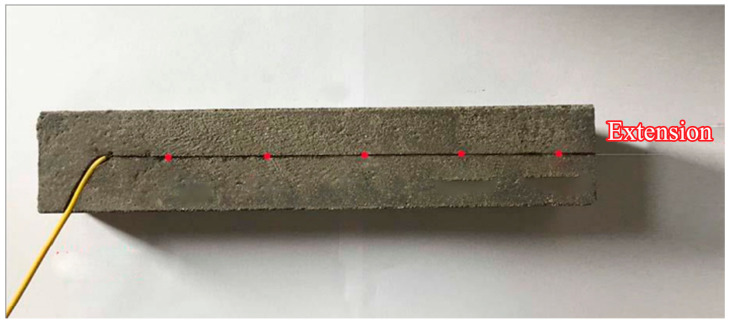
FBG sensor installation.

(1) Based on the composition and mix ratio of the sandstone-like material for microfissured specimens, precisely weigh sand, cement, iron concentrate powder, and water.
(2) Thoroughly mix the measured cement, sand, and iron concentrate powder in a mixer.
(3) After homogenizing the solid materials, add the pre-weighed water to the mixer and mix for 2 min. Subsequently, incorporate a water reducer and continue mixing for an additional 2 min.
(4) Assemble the first mold set by replacing its spacer plates with a JRC = 10–12 pair. Coat the inner walls uniformly with mechanical lubricant. Fill the mold with the prepared concrete mixture, then vibrate the mold on a shaking table for 1 min. Repeat filling and vibration until the mixture aligns flush with the mold surface. Level the upper surface and seal it with plastic film, as shown in Figure 7. After casting Specimens 1-1 and 1-2, cure them at 20 °C ± 2 °C and 95% relative humidity for 24 h. Demold and continue curing for 7 days.

(5) Select Specimen 1-1 from Step (4). Machine a groove on its roughened surface to accommodate an FBG sensor. The groove width is 1–2 times the fiber diameter, and its depth equals the fiber diameter. Secure the FBG sensor in the groove using adhesive, as shown in Figure 8.

**Figure 9:**
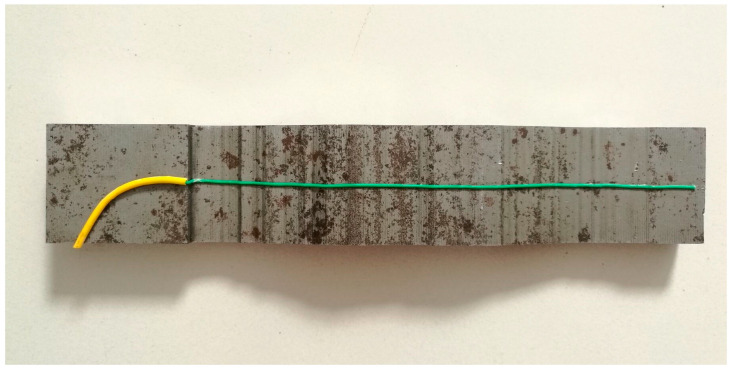
Precast groove formation.

To ensure groove quality, a metal wire (slightly thicker than the fiber diameter) is pre-attached to the spacer plate surface. During initial specimen casting, this wire creates a uniform groove for FBG fixation, as shown in Figure 9.

**Figure 10:**
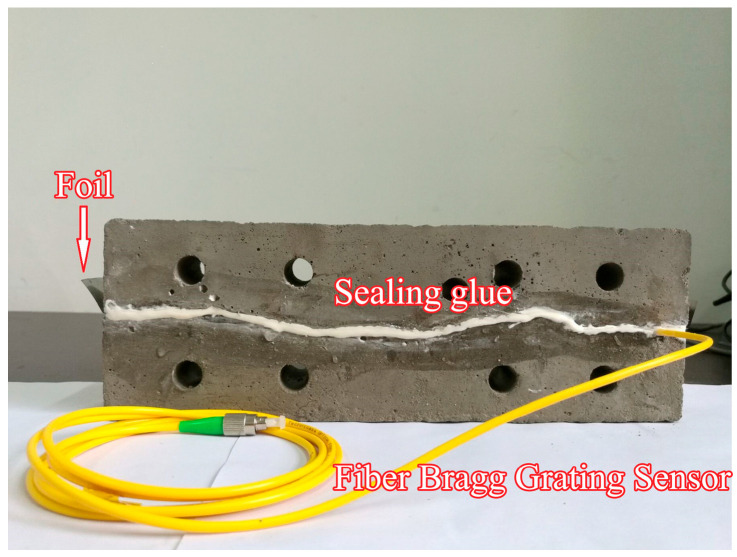
Core specimen.

**Figure 11:**
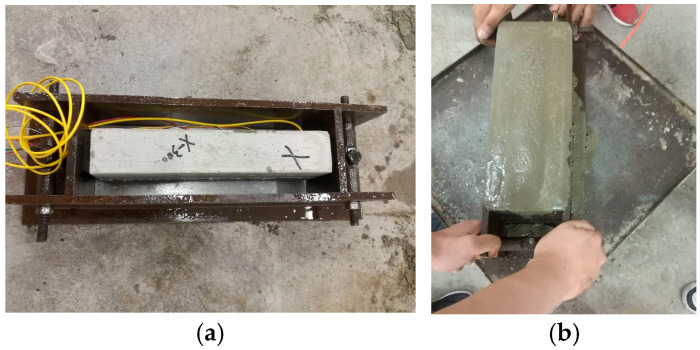
Grout encapsulation: (**a**) core positioning; (**b**) vibration compaction.

**Figure 12:**
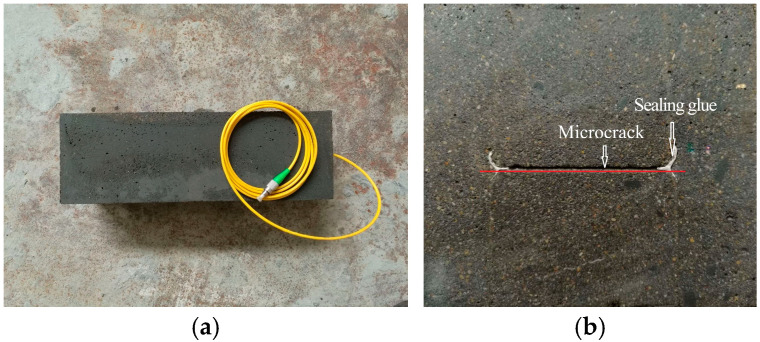
Grouted specimen with sandstone-like microfissures: (**a**) top view of the specimen; (**b**) cross-section view of the specimen.

(6) Position Specimen 1-2 onto the modified Specimen 1-1 from Step (5), ensuring full coupling of their roughness profiles to assemble the core specimen.
(7) Insert 200 μm-thick steel shims into gaps at both ends of the core specimen to create a uniform microfissure matching the shim thickness. Seal lateral gaps with waterproof adhesive to secure the specimens and prevent grout infiltration during subsequent casting. The shims penetrate 3–5 cm into the microfissure, with widths matching the specimen dimensions. The core specimen configuration is shown in Figure 10.

(8) Prepare the second mold set by uniformly coating its inner walls with mechanical lubricant. Place the processed core specimen from Step (7) into the mold, routing exposed FBG sensor cables through the sensor lead-through holes on the insert plate, as shown in Figure 11.

(9) Following steps (1) and (2), prepare fresh concrete grout. Fill the second mold with the mixture, vibrate on a shaking table for 30 s, and repeat filling/vibration cycles until the grout aligns flush with the mold surface. Level the top surface and cover it with plastic film.
(10) After 24 h of curing at 20 °C ± 2 °C and 95% relative humidity, demold the specimen. Remove the steel shims and continue curing for an additional 28 h under identical conditions. This completes the fabrication of the sandstone-like grouting test block containing a 200 μm-aperture microfissure with JRC = 10–12, as shown in Figure 12a.

Preliminary trials were conducted following the above procedures. Cross-sectional analysis of pre-prepared specimens, as shown in Figure 12b, confirmed intact microfissure geometry and effective sealing by the adhesive. The fabricated grouting specimens meet experimental requirements for subsequent testing.

### 2.3. Grouting Test System

**Figure 13:**
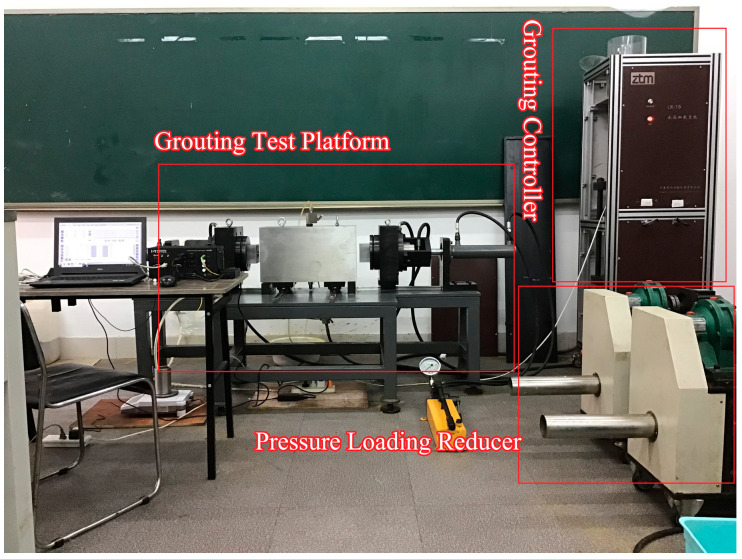
Triaxial grouting simulation system schematic.

The triaxial stress grouting simulation test system for microfissured specimens comprises four subsystems: (1) triaxial stress grouting test platform, (2) triaxial stress loading system, (3) grout control system, and (4) data acquisition system. The overall configuration of the microfissured triaxial stress grouting-seepage test system is illustrated in Figure 13.

**Figure 14:**
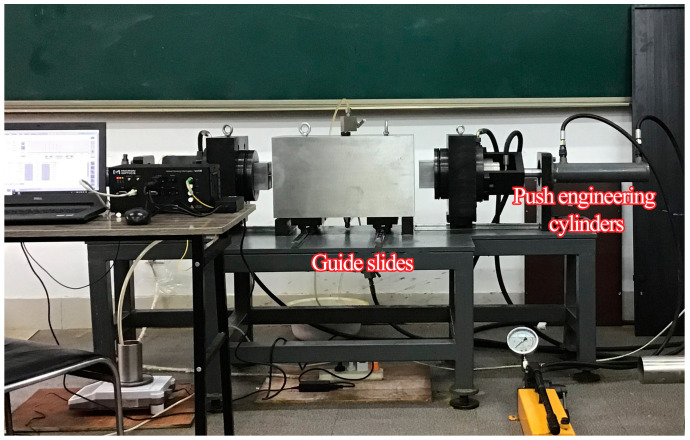
Mobile platform with axial/confining pressure chambers.

The triaxial stress grouting test platform shown in Figure 14 comprises a support frame, test chamber, axial stress loading pistons, and hydraulic drive cylinders. The support frame integrates two pairs of guiding sliding devices: one pair enables lateral movement of the axial stress pistons via hydraulic actuation, while the other facilitates front-rear positioning of the confining pressure chamber.

**Figure 15:**
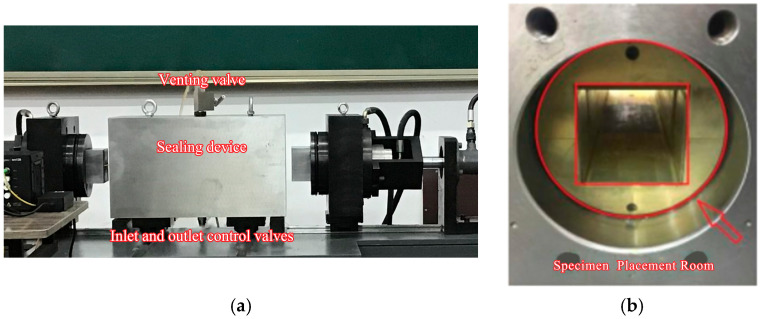
Cross-section of pressure-resistant grout cell: (**a**) test box; (**b**) specimen placement room.

The microfissure grouting test chamber primarily consists of a sealing assembly, confining pressure chamber, specimen housing compartment, specimen sealing sleeve, and embedded axial pistons at both ends. The sealing assembly incorporates an exhaust valve at its upper section and confining pressure fluid inlet/outlet control valves at the lower section. These control valves interface with the confining pressure chamber through dedicated fluid ports within the sealing assembly. The confining pressure chamber, housed within the sealing assembly’s inner cavity, combines with the specimen sealing sleeve to collectively create the specimen housing compartment, as shown in Figure 15.

**Figure 16:**
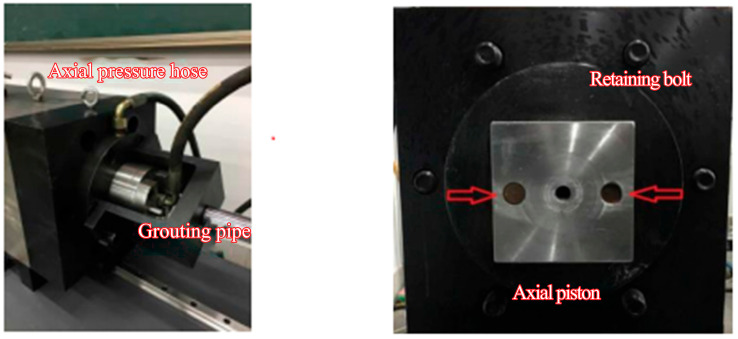
Axial piston with integrated grout channels.

The axial stress pistons for microfissure grouting tests shown in Figure 14 consist of axial loading pistons, hydraulic cylinders, and axial seal cylinder caps, symmetrically arranged on both sides of the test platform. Each axial loading piston, as shown in Figure 16, is equipped with communication channels for sensor cabling and grout inlet/outlet ports connected to the grouting pump via injection tubing, while the hydraulic cylinders are linked to the axial loading pistons through oil lines. Operationally, the confining pressure chamber is positioned centrally, and hydraulic drive cylinders advance the axial pistons at both ends, embedding the axial loading pistons into the specimen housing compartment and inserting the hydraulic cylinder ends into the sealing assembly’s inner cavity. Sealing is achieved via fastening nuts on the axial seal caps. The integrated system—comprising the confining pressure chamber, specimen sealing sleeve, and bilateral axial pistons—forms a sealed specimen housing compartment. Together with the grouting-seepage axial pistons, this assembly creates a closed triaxial stress chamber capable of simulating in situ grouting conditions under controlled three-dimensional stress states.

**Figure 17:**
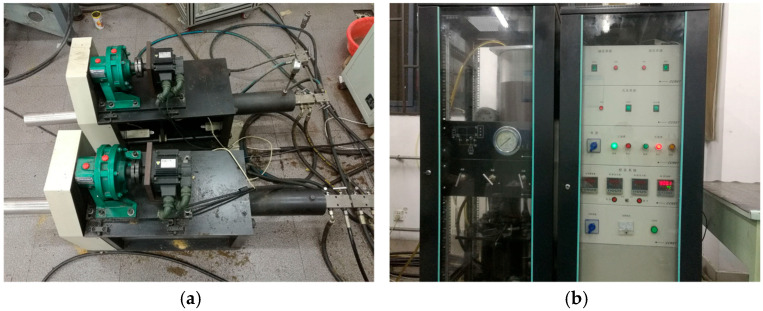
Axial and peripheral pressure loading systems: (**a**) cycloidal pinwheel reducer; (**b**) stress loading control system.

The triaxial stress loading system comprises a stress control unit and two cycloidal gear reducers. One reducer supplies confining pressure via fluid lines connected to the inlet valve of the grouting-seepage test chamber, while the other delivers axial stress through hydraulic cylinders linked to the axial pistons. The stress control unit regulates both the magnitude and loading rate of triaxial stresses, as shown in Figure 17.

**Figure 18:**
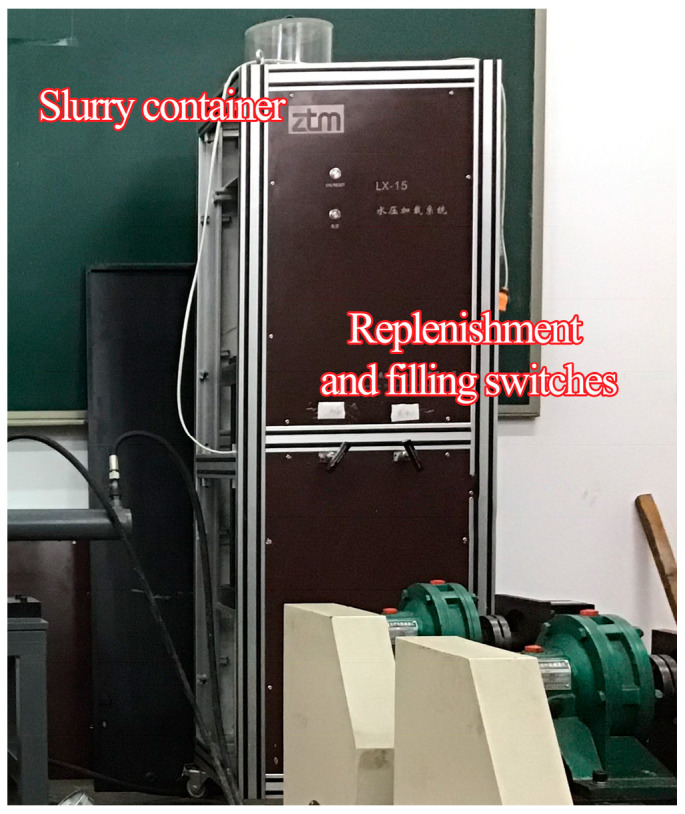
Grout pressure regulation assembly.

The grout control system shown in Figure 18 integrates a slurry tank, a grouting pump, and a differential pressure regulator. The pump connects to the grout ports of the triaxial stress-seepage test platform through pressure-rated tubing, with grouting pressure precisely modulated via the control unit.

**Figure 19:**
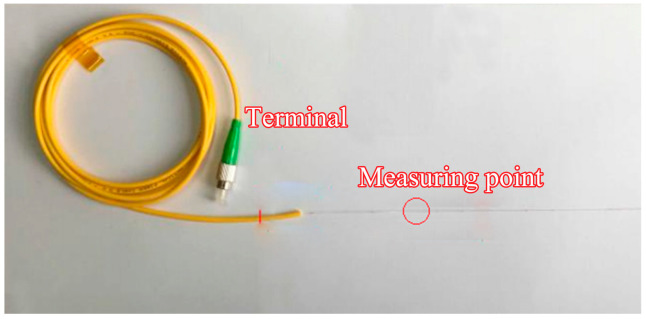
FBG sensor configuration with protective sheathing.

The data acquisition system employs FBG sensors pre-embedded within the grouting test blocks. Tailored to the characteristics of microfissured sandstone specimens and experimental requirements for grouting-seepage studies, the FBG sensor configuration is illustrated in Figure 19. Each sensor array incorporates four measurement nodes spaced at 70 mm intervals along a bare fiber segment, with polymer-coated protective sheathing applied to non-monitored regions. Signal processing equipment records real-time strain data during testing.

### 2.4. Test Loading Process

In accordance with the design specifications of the sandstone microfissure grouting simulation tests presented in this study, three sets of grouting test blocks with varying roughness coefficients (JRC = 0–2, 4–6, and 10–12) were fabricated, each featuring a designed microfissure aperture of 200 μm. Based on in situ stress and seepage field conditions encountered in deep mine surrounding rocks, the grouting tests were conducted under confining pressure levels of 6 MPa and 7 MPa.

The grout used in this grouting test was an ultrafine cement slurry prepared according to the mix proportion of W/C ratio = 1.4.

**Figure 20:**
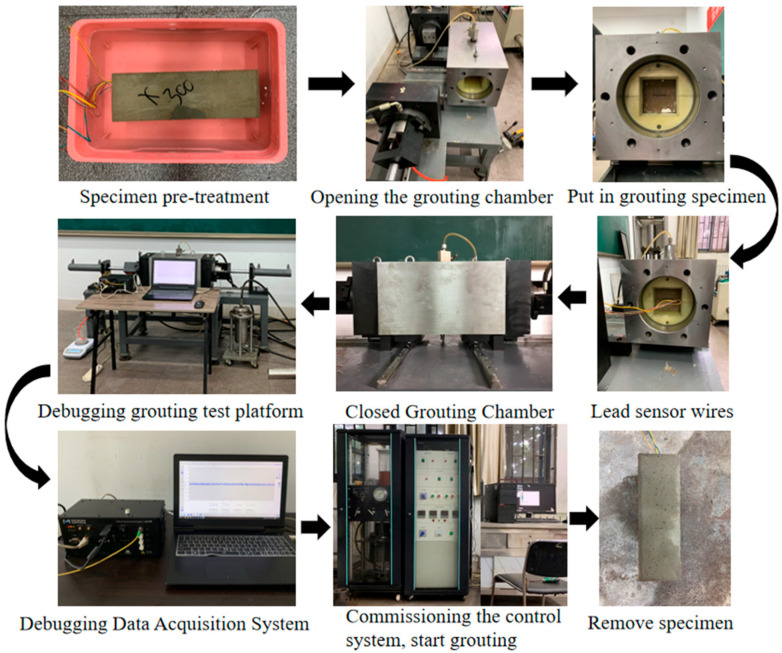
Workflow of sandstone microfissure grouting simulation.

The workflow for the sandstone microfissure grouting simulation tests is shown in Figure 20.

The load application procedure in grouting simulation tests comprises the following steps:

(1) Apply a 0.5 MPa axial preload to ensure full contact between the axial pistons and specimen ends. Subsequently, incrementally increase confining and axial pressures in 0.5 MPa steps until reaching target confining stress levels.
(2) Reset monitoring data on the FBG interrogator to eliminate strain effects induced by initial stress application.
(3) Initiate grouting at 1.0 MPa pressure. Upon stabilization of grout flow, record strain values at all measurement nodes within the microfissure under this pressure condition, with data acquisition spanning a 60 s interval. Progressively elevate grouting pressure in 1.0 MPa increments until reaching the predefined limit. This protocol enables systematic investigation of sandstone microfissure grouting behavior under constant confining pressure with varying grouting pressures.

## 3. Experimental Results and Discussion

### 3.1. Ultrafine Cement Particle Size and Viscosity Analysis

The particle size and viscosity of cement grains critically influence grouting efficacy in microfissured media. To select optimal ultrafine cement grout for sandstone microfissure grouting simulations, this study first characterized the particle size distribution of candidate cement materials. Subsequently, five grout formulations with varying W/C ratios were systematically designed to evaluate key performance parameters, including viscosity and initial setting time, enabling identification of the optimal mix for experimental requirements.

#### 3.1.1. Analysis of Ultrafine Cement Particle Size Test Results

**Figure 21:**
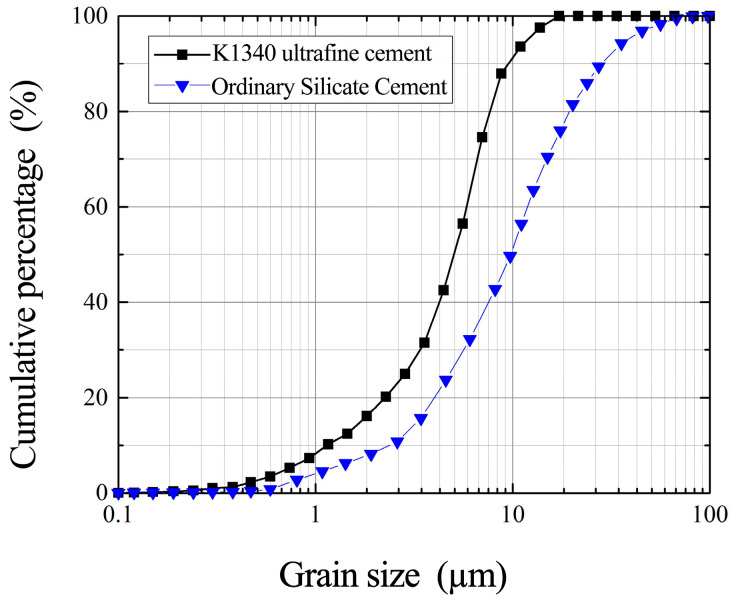
Cumulative particle size distribution of K1340 ultrafine cement.

Cement particle size directly governs grout injectability into microfissures, serving as a pivotal factor in sandstone microfissure grouting modeling. Particle size analysis was conducted using an LS900 laser particle size analyzer, with the resulting size distribution visualized in Figure 21.

As shown in Figure 21, the ultrafine cement exhibits particle sizes of D_50_ = 5.187 μm, D_75_ = 7.90 μm, and D_90_ = 10.69 μm, with the majority of particles concentrated within the range of 4.91 μm to 10.69 μm. Considering particle aggregation due to hydration and the clogging effects of particle clusters, the Mitchell formula was applied to determine the minimum injectable microfissure aperture for the grout. The maximum particle sizes Dmax = 14 μm and D_95_ = 12 μm were selected for this analysis.

They are judged according to Mitchell’s formula, written as follows:

(1) $$\begin{array}{l} D_{f1}=GR\cdot D_{\max}\geq 3\times 14=42\\ D_{f2}=GR\cdot D_{95}\geq 5\times 12=60 \end{array}$$

From Equation (1), the ultrafine cement slurry can theoretically be injected into rock microfissures with openness greater than 60 μm, which can meet the requirements of the sandstone microfissure grouting simulation test in this paper.

#### 3.1.2. Viscosity Test Result Analysis

Grout viscosity quantifies the internal frictional resistance during fluid flow, with lower viscosity corresponding to reduced flow resistance and enhanced mobility/injectability. As the W/C ratio critically governs viscosity, systematic viscosity measurements were conducted on ultrafine cement grouts with W/C ratios of 1.0, 1.2, 1.4, and 1.6.

**Figure 22:**
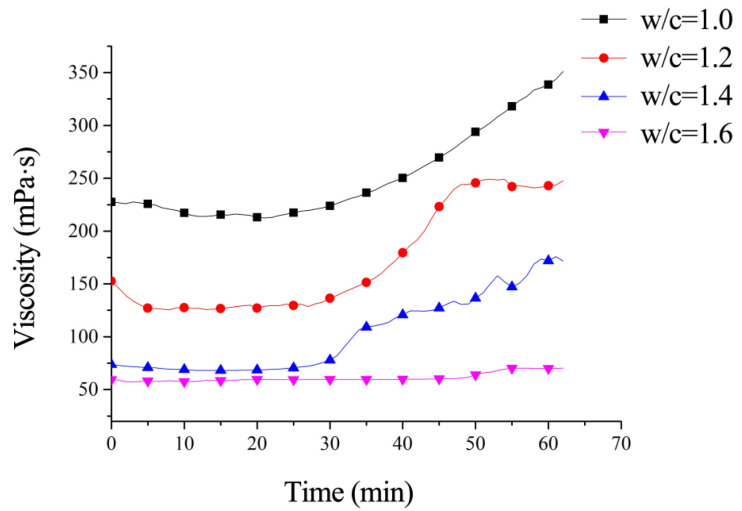
Viscosity evolution of ultrafine cement grouts.

Experimental testing utilized an SNB-2 digital viscometer (manufactured by a Shanghai-based instrument company, Shanghai, China) to evaluate time-dependent viscosity variations. The temporal evolution of viscosity for each grout formulation is shown in Figure 22.

As shown in Figure 22, the viscosity of ultrafine cement grout decreases significantly with increasing W/C ratio. Grout with a W/C ratio = 1.0 exhibits an initial viscosity approximately fourfold higher than that of grout with a W/C ratio = 1.6. When the W/C ratio exceeds 1.4, the influence of further ratio increases on viscosity diminishes significantly. Time-dependent analysis reveals stable viscosity growth within the first 30 min, followed by accelerated viscosity increase thereafter. Notably, the grout with W/C ratio = 1.6 exhibited no significant viscosity increase over time. For grout with W/C ratio = 1.4, the viscosity stabilized at approximately 75 mPa·s during the initial 30 min period. According to the analysis by Qiao et al. [30], microfissures with apertures greater than 0.07 mm can theoretically be injected.

### 3.2. Injectability Analysis of Sandstone Microfissures

**Figure 23:**
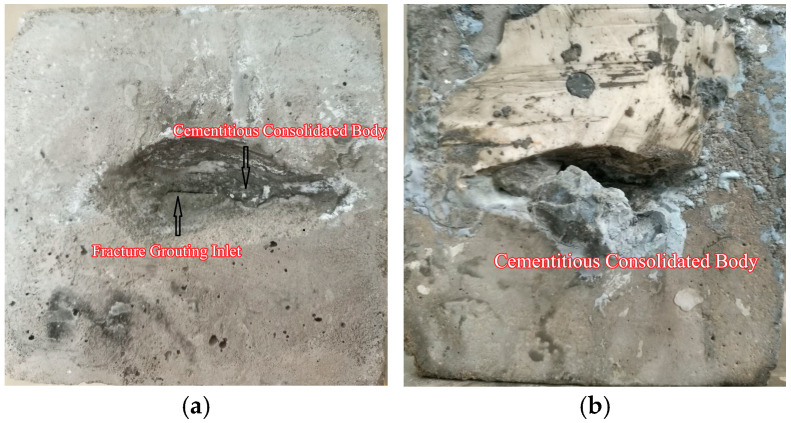
Grout inlet morphology after grouting in sandstone-like microfissure specimens: (**a**) grouting inlet of fracture; (**b**) grout consolidation body.

**Figure 24:**
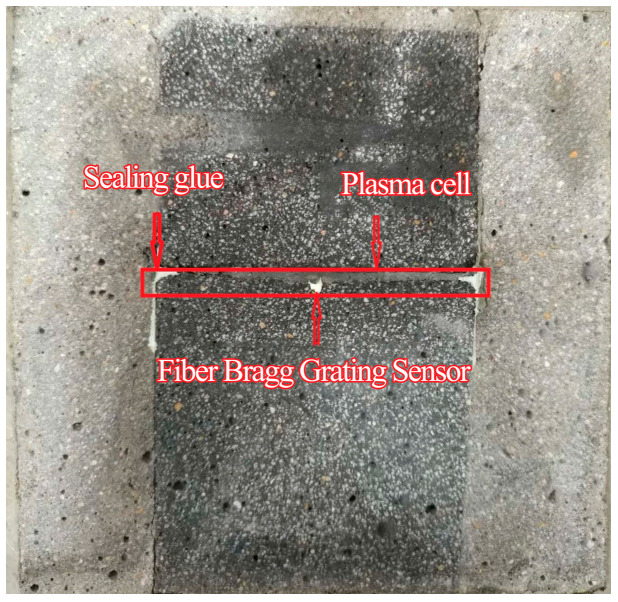
Cross-sectional grout distribution 15 cm from injection point.

Grouting simulations were conducted on microfissured sandstone-like specimens with three distinct roughness levels (JRC = 0–2, 4–6, 10–12) and an initial aperture of 0.2 mm under varying confining pressures. Post-grouting specimens were sectioned to evaluate the sealing efficacy of ultrafine cement grout. The grout inlet morphology after grouting in sandstone-like microfissure specimens is shown in Figure 23. The cross-section of the specimen after grouting is shown in Figure 24.

Grout inlet morphology is shown in Figure 23. Post-grouting analysis of microfissured sandstone-like specimens revealed significant accumulation of ultrafine cement grout stone bodies at injection inlets, leading to partial sealing. This clogging phenomenon is attributed to a “filter screen effect” at the narrow microfissure entrance: larger particle aggregates within the grout are intercepted and deposited as the slurry passes through the constricted aperture. Progressive particle deposition gradually obstructs the microfissure entrance. Experimental observations confirmed that increased microfissure roughness (e.g., JRC = 10–12 specimen in Figure 23b) exacerbates particle aggregation, markedly reducing grouting success rates due to severe inlet blockage.

Cross-sectional analysis shown in Figure 24, under stepwise grouting pressure increments over a 15 min duration, and cross-sectional examination at 15 cm from the injection inlet demonstrated dense grout stone body distribution and effective microfissure sealing. The uniform penetration and robust interfacial bonding between the grout and sandstone matrix validate the sealing efficacy under controlled triaxial stress conditions.

### 3.3. Seepage Dynamics Under Varied Roughness Conditions

**Figure 25:**
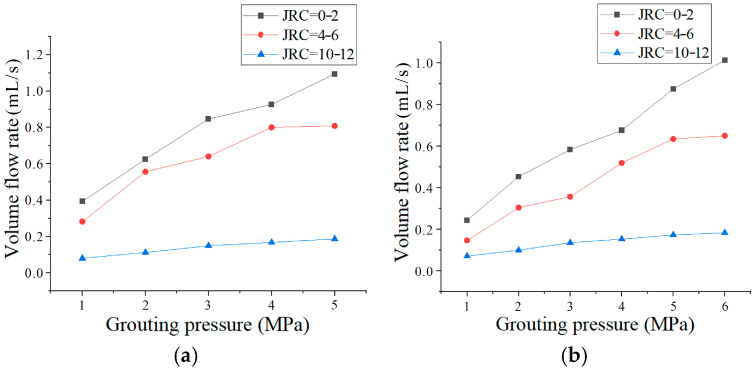
Volumetric flow rate vs. injection pressure under different roughness conditions: (**a**) 6 MPa confining pressure; (**b**) 7 MPa confining pressure.

Following the experimental design, grouting tests were conducted on sandstone microfissures with an initial aperture of 0.2 mm and roughness coefficients JRC of 0–2, 4–6, and 10–12 under confining pressures of 6 MPa and 7 MPa. Grouting pressure was incrementally increased from 1 MPa to predefined limits. Volumetric flow rates under varying grouting pressures and confining conditions were obtained through real-time grout volume monitoring. The relationship between volumetric flow rate and grouting pressure for specimens with different roughness levels under distinct confining pressures is illustrated in Figure 25.

As shown in Figure 25, the grout flow rate exhibits a nonlinear functional dependence on grouting pressure. Under identical confining and injection pressures, the grout volumetric flow rate decreases with increasing roughness. Specifically, JRC = 0–2: the flow rate increases progressively with rising injection pressure; JRC = 4–6: flow rate also increases with pressure, but the growth rate diminishes at higher pressures; and JRC = 10–12: minimal flow rate variation occurs despite pressure increments due to enhanced flow resistance and particle clogging. At 7 MPa confining pressure, a notable flow rate surge is observed when injection pressure escalates from 3 MPa to 4 MPa (Figure 25b). This is attributed to the disruption of deposited cement agglomerates, which reopens clogged flow channels. For high-roughness specimens (JRC = 10–12), elevated surface irregularities amplify flow resistance, promoting particle retention at asperities. Subsequent pressure increases fail to remobilize these anchored particles, resulting in limited flow rate improvement despite higher injection pressures.

Comparison of Figure 25a,b reveals reduced flow rates under higher confining pressures for specimens of identical roughness. This is due to aperture compression under increased confinement, which elevates flow resistance. The effect is magnified in high-JRC specimens, where roughness-induced tortuosity further restricts grout penetration.

In engineering applications, (1) Grout pressure limits: The high roughness microfissure (JRC = 10–12) tends to be saturated with flow growth at >4 MPa pressure. Therefore, when the flow gain due to pressure boosting is less than 20% of the initial value (e.g., only 0.05 mL/s at 4→5 MPa in the JRC = 10–12 zone), the boosting should be stopped to avoid ineffective energy consumption. (2) Roughness threshold: The flow rate decreases sharply at JRC > 8 (Figure 25 curve slope change point), and JRC = 8.5 is determined to be the critical value of grouting failure. (3) Clogging mitigation: In response to the flow decay phenomenon, a “pressure oscillation decongestion” mechanism was designed: a pulsed pressure fluctuation with an amplitude of 0.5 MPa (frequency of 0.2 Hz) was implemented at the critical point of the flow decline, destroying the depositional skeleton through shear force perturbation.

According to the empirical model proposed by Zhang et al. [31], the hydraulic gradient J is defined as follows:

(2) $$J=\frac{\Delta Ρ}{\rho gL}$$

where Δ*P* represents the pressure difference (MPa) between the grout inlet and outlet, *ρ* is the grout density (1.38 g/cm^3^), and *L* denotes the vertical distance (0.3 m) between the specimen boundaries.

**Figure 26:**
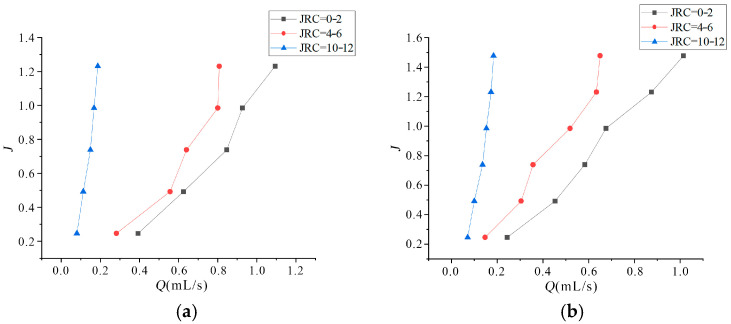
Relationship between J and Q under different roughness conditions: (**a**) 6 MPa confining pressure; (**b**) 7 MPa confining pressure.

Based on grouting test data, the relationship between hydraulic gradient (J) and volumetric flow rate (Q) under varying roughness conditions was calculated, as shown in Figure 26.

Experimental observations revealed that the Q of grout within microfissures exhibits a marked increase with rising J, as shown in Figure 26. When the surface roughness of microfissures progressively increases, a higher hydraulic gradient is required to maintain identical flow rates, demonstrating the dominant influence of wall morphology on flow resistance. The quadratic relationship between hydraulic gradient and volumetric flow rate indicates that the seepage-diffusion behavior of ultrafine particle grout in microfissured media deviates from the Darcian linear flow regime. To address this, the Forchheimer nonlinear flow model [32,33,34] was adopted to establish a constitutive equation that accurately characterizes the seepage mechanics of ultrafine cement grout in microfissures, written as follows:

(3) $$J=aQ+bQ^{2}$$

where a is viscous resistance coefficient and b is inertial resistance coefficient.

Regression analysis of the experimental data in Figure 25 using Equation (3) yielded the fitted curves shown in Figure 26, with corresponding Forchheimer coefficients detailed in Table 3.

Mechanistic Interpretation: viscous dominance (a) governs low-velocity flow through tortuous paths; inertial dominance (b) dictates high-velocity particle-fluid interactions; and roughness amplification indicates a and b increase exponentially with JRC (R^2^ > 0.95). This model effectively captures the coupled viscous-inertial dynamics governing ultrafine cement grout permeation in rough-walled microfissures. Compared coefficients a and b with those reported in the Ph.D. theses by Sun [35], Jiang [36], and Ren [37], all of which investigated nonlinear flow in fractured rock masses: (1) compared to Sun [35], our viscous resistance coefficient a is substantially lower, driven by reduced fracture roughness (JRC) and distinct geostress conditions; (2) compared to Jiang [36], their inertial resistance coefficient b is markedly diminished due to viscosity reduction via graphene modification; and (3) compared Ren [37], the nonlinear threshold (b/a) is approximately lower owing to fracture network effects in fault zones. These quantitative contrasts enhance the engineering context.

### 3.4. Deformation Characteristics of Microfissures with Varying Roughness

**Figure 27:**
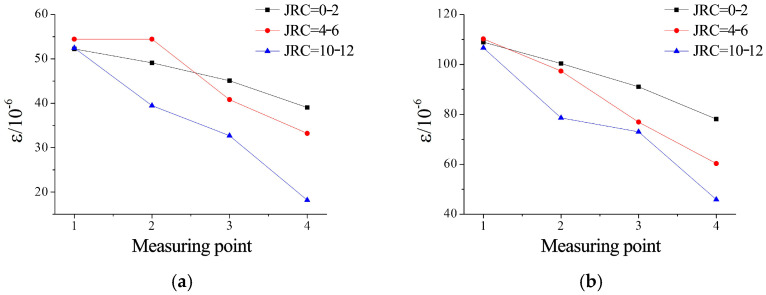
Strain values at measurement points under different roughness conditions: (**a**) confining pressure of 7 MPa with grouting pressure of 2 MPa; (**b**) confining pressure of 7 MPa with grouting pressure of 4 MPa.

**Figure 28:**
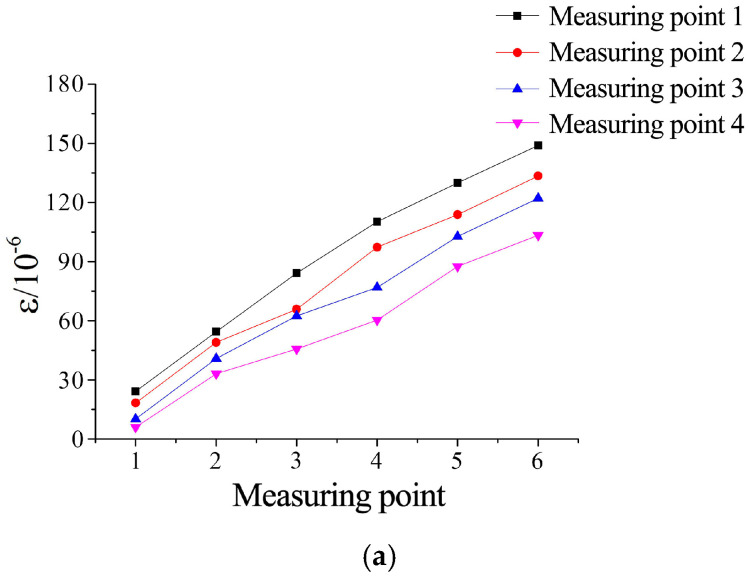

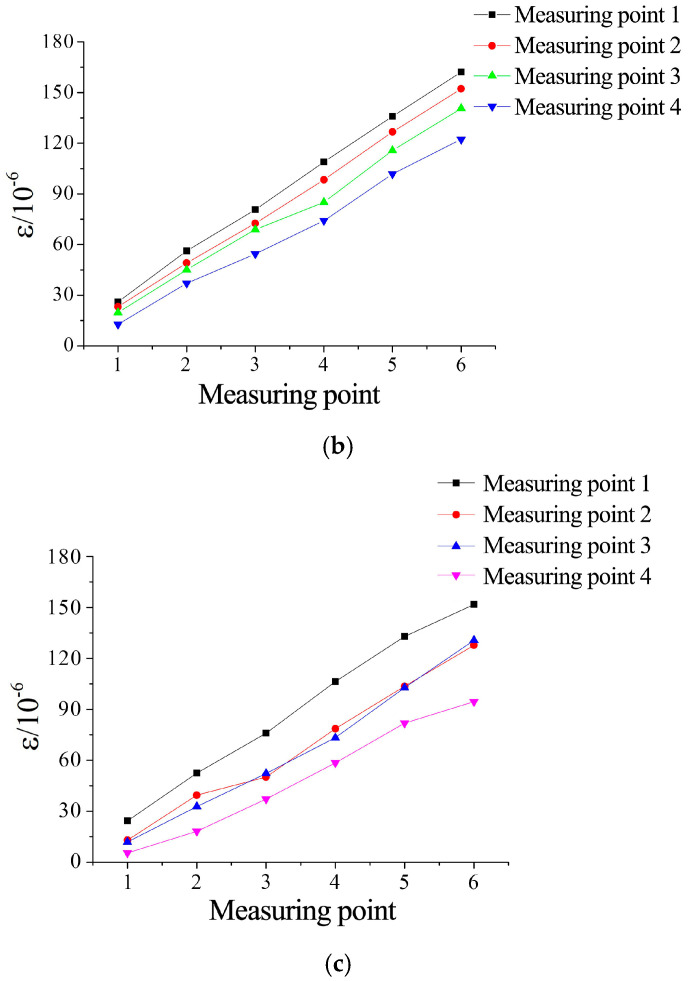
Strain values under different grouting pressures for distinct roughness levels: (**a**) JRC = 0–2, (**b**) JRC = 4–6, and (**c**) JRC = 10–12.

**Figure 29:**
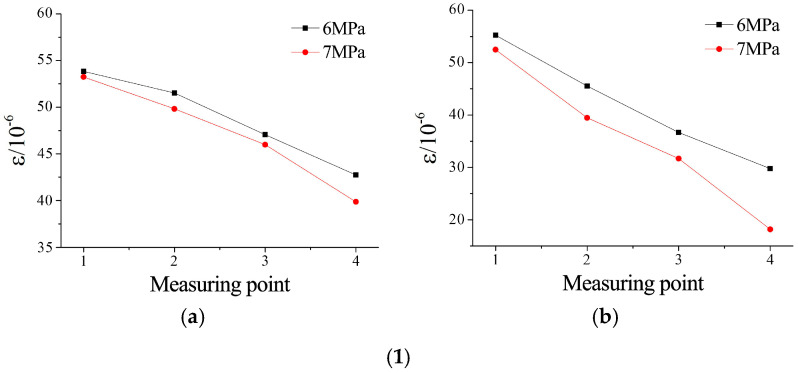

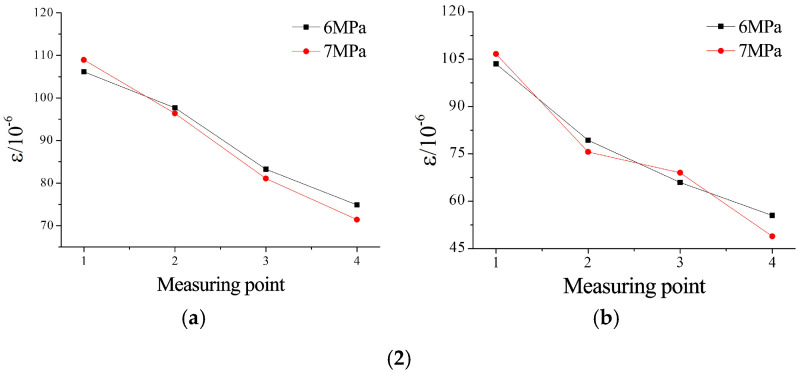
Strain variations under different confining pressures: (**1**) grouting pressure of 2 MPa: (**a**) JRC = 0–2 and (**b**) JRC = 10–12; (**2**) grouting pressure of 4 MPa: (**a**) JRC = 0–2 and (**b**) JRC = 10–12.

FBG sensors pre-embedded within microfissured grouting specimens were employed to monitor strain variations induced by ultrafine cement grout infiltration during testing. Strain data under varying confining pressures and grouting pressures were recorded and analyzed. Strain distributions at measurement points under different confining and grouting pressure conditions are illustrated in Figure 27, Figure 28 and Figure 29.

Strain evolution analysis shown in Figure 27: under a constant confining pressure of 7 MPa, the relative strain differences among measurement points increased with higher microfissure roughness. For JRC = 0–2, strain values decreased systematically with distance from the grouting inlet. At JRC = 4–6, localized strain magnitudes exceeded those of JRC = 0–2 under identical pressures, yet exhibited accelerated attenuation rates. For JRC = 10–12, the strain differential between the first and last measurement points reached 3.5 times that of JRC = 0–2. Notably, minimal strain variation occurred between the second and third measurement points, as shown in Figure 27b, attributed to pressure concentration effects near surface asperities at the third sensor location.

Pressure-dependent strain behavior is shown in Figure 28. For all roughness levels, strain at the first measurement point increased linearly with grouting pressure. This uniformity stems from a 50 mm parallel-smooth transition microfissure intentionally created near the inlet during specimen fabrication. At JRC = 0–2, strain increments across all measurement points followed consistent trends with pressure escalation. Higher roughness (JRC = 4–12) induced complex nonlinear strain responses at downstream measurement points. Specifically, for JRC = 10–12, minimal strain variation between the second and third points suggests that enhanced roughness amplifies flow resistance heterogeneity, altering the effective resistance angle during grout propagation.

Analysis of Figure 29, at a grouting pressure of 2 MPa, showed that increased confining pressure induced slight reductions in strain at identical measurement points. Comparative analysis of Figure 29(1,2) reveals that specimens with higher roughness exhibited greater sensitivity to confining pressure variations. This phenomenon is attributed to confining pressure-induced reduction in the initial aperture of microfissures, thereby increasing flow path resistance. As grouting pressure escalated to 4 MPa (Figure 29(1) vs. Figure 29(2)), the influence of confining pressure on strain responses diminished significantly.

## 4. Conclusions

A triaxial stress-seepage grouting mechanism test system for microfissures was utilized to investigate the injectability and deformation characteristics of sandstone microfissures with initial apertures of 200 μm and varying roughness levels (JRC = 0–2, JRC = 4–6, JRC = 10–12). Grouting simulations were conducted under confining pressures of 6 MPa and 7 MPa with different grouting pressures. The following conclusions were drawn:

(1) A novel method for preparing monitorable microfissure seepage specimens was proposed, enabling the fabrication of microfissured grouting specimens with preset apertures and roughness. By embedding FBG sensors within the specimens, real-time monitoring of microfissure surface deformation was achieved.
(2) Experimental results indicate particle aggregation and deposition of ultrafine cement grout at microfissure inlets due to filtration effects, where larger aggregates are intercepted. Increasing surface roughness intensified deposition, reducing grouting simulation success rates. Cross-sectional analysis confirmed subsequent formation of dense cementitious bodies, achieving effective sealing.
(3) Under identical confining and grouting pressures, ultrafine cement grout volume flow decreases with increasing roughness, while increasing grouting pressure elevates flow rates. This is attributed to pressure disrupting deposited particles and reopening flow channels. The rate of flow velocity increase diminishes with higher roughness.
(4) For JRC = 10–12, increased grouting pressure exhibits limited influence on seepage behavior. This is attributed to enhanced flow resistance caused by rough microfissure surfaces, facilitating localized particle accumulation and blockage. Subsequent pressure increments prove insufficient to disperse deposits accumulated in regions with large resistance angles.
(5) Under constant grouting pressure, the relative strain variation between measurement points increases with roughness. Complex strain responses at secondary measurement points (e.g., points 2 and 3) under varying pressures suggest that heightened roughness amplifies the fluctuation range of normal resistance angles during grout flow.

Under identical grouting pressure conditions, as the roughness of microfissure specimens increases, the relative variation of strain values between measurement points gradually increases. With increasing fissure roughness, the variation patterns of strain with grouting pressure at the second and third measurement points become more complex. Analysis indicates that increased fissure roughness leads to a wider variability range in the normal resistance angle during grout flow within fissures.

Although the grouting specimen prepared in the grouting test in this paper considered the effect of roughness, it was a single-microfissure model, and in the actual rock body, the microfissure exists in the form of cross-microfissures and microfissure networks, so the preparation method of the specimen at a later stage should take into account the effect of multiple microfissures. The room temperature during this test was constant, ignoring the potential effect of the ground temperature gradient, while the short-term test may not be able to capture the evolution of microfissure behavior due to, e.g., chemical precipitation and creep deformation. In this indoor microfissure grouting test, the microfissure triaxial stress grouting system was utilized to achieve the adjustment of the perimeter pressure and grouting pressure applied to the test block, but it could not achieve the observation of the flow state of the slurry, and the research and development of the visual grouting test system should be strengthened, which can more intuitively study the microfissure grouting process.

In practical engineering, tiered grouting should be implemented according to the permeability of different strata to enhance on-site grouting efficiency; due to the complexity of rock mass geological conditions, composite grouting materials with good stability, effective permeability, and minimal environmental pollution are continuously being developed to further improve grouting precision and efficacy.

## Figures and Tables

**Table 1:** Physical and mechanical properties of K1340 ultrafine Portland cement.

Setting Time		Compressive Strength/MPa		Flexural Strength/MPa	
Initial Setting Time/min	Final Setting Time/min	3d	28d	3d	28d
≥30	≤600	23	52.5	4	7

**Table 2:** Physical and mechanical properties of ordinary Portland cement.

Density/kg·m^−3^	Compressive Strength/MPa	Flexural Strength/MPa	Setting Time	
			Initial Setting Time/mm	Final Setting Time/mm
3000	47.5	8.7	115	310

**Table 3:** Forchheimer coefficients derived from nonlinear regression.

Confining Pressure (MPa)	Microfissure Opening (μm)	Axial Pressure (MPa)	JRC	a	b	R^2^
6	200	6	0–2	0.309	0.751	0.99
			4–6	0.240	1.399	0.95
			10–12	0.546	31.723	0.99
7		7	0–2	0.997	0.476	0.99
			4–6	1.492	0.948	0.97
			10–12	0.515	39.257	0.99

## Data Availability

The original contributions presented in this study are included in the article/Supplementary Material. Further inquiries can be directed to the corresponding author.

## Supplementary Materials

The following supporting information can be downloaded at: https://www.mdpi.com/article/10.3390/ma18163746/s1, Figure S1: Flow chart of the method of preparing the monitoring microfissure grouting sample; Figure S2: Preparation of similar sandstone materials: (a) Prepare materials, (b) Mix until homogeneous.; Table S1: Contrast table of ultrafine cement particle size.

## Abbreviations

The following abbreviations are used in this manuscript:

FBG	Fiber Bragg grating
JRC	Joint roughness coefficient
