# Supplementary material for: Experimental Study on Grouting Seepage Characteristics in Rough Single Microfissure Under Triaxial Stress States
Source: Materials (Basel). 2025 Aug 11;18(16):3746. doi: 10.3390/ma18163746 (PMC12387365; doi:10.3390/ma18163746)
Supplement: Supplementary file 1 [file materials-18-03746-s001.zip › materials-3697323.pdf]

The supplementary figures and table are provided below:

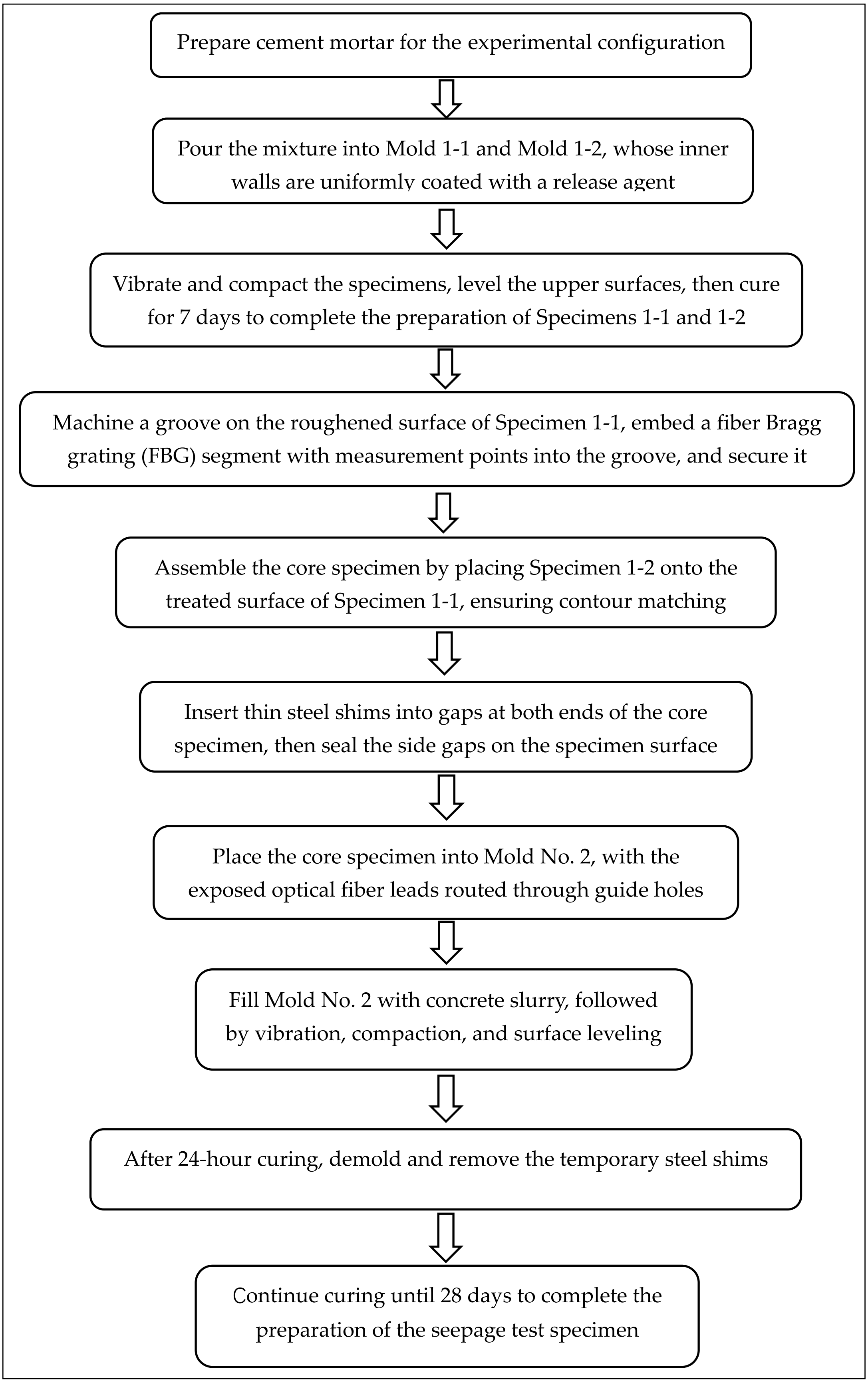

Figure S1 Flow chart of the method of preparing the monitoring microfissure grouting sample

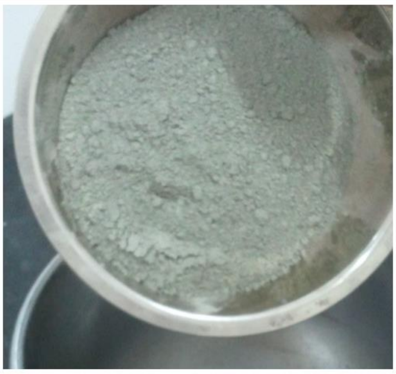

(a)

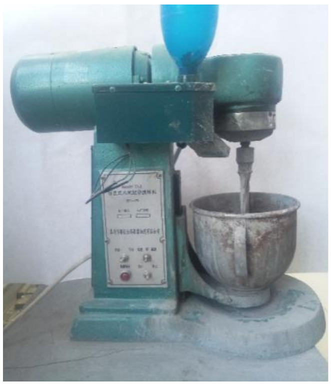

(b)

Figure S2 Preparation of similar sandstone materials: (a) Prepare materials, (b) Mix until homogeneous.

Table S1 Contrast table of ultrafine cement particle size

**Sample**	**Replicates**	**D_{50}/μm**	**D_{75}/μm**	**D_{90}/μm**	**D_{max}/μm**	**Mean D_{50}/μm**	**Mean D_{75}/μm**	**Mean D_{90}/μm**
K1340	1	5.32	7.81	10.41	14.00	5.19	7.90	10.69
	2	4.97	8.03	11.06	14.00			
	3	5.28	7.88	10.61	14.00			
